# DDX3X Promotes Rotavirus Infection and Serves as an Antiviral Target

**DOI:** 10.1155/tbed/5822897

**Published:** 2025-11-21

**Authors:** Pengfei Hao, Yuchen Liu, Chunmei Cui, Letian Li, Qiaoqiao Qu, Limin Shang, Jing Chen, Yuhang Jiang, Ronglan Yin, Jian Wang, Guoqing Wang, Chang Li

**Affiliations:** ^1^State Key Laboratory for Diagnosis and Treatment of Severe Zoonotic Infectious Diseases, Key Laboratory of Zoonosis Research, Ministry of Education, College of Basic Medical Science, Jilin University, Changchun, China; ^2^State Key Laboratory of Proteomics, Beijing Proteome Research Center, National Center for Protein Sciences (Beijing), Beijing Institute of Lifeomics, Beijing, China; ^3^Research Unit of Key Technologies for Prevention and Control of Virus Zoonoses, Chinese Academy of Medical Sciences, Changchun Veterinary Research Institute, Chinese Academy of Agricultural Sciences, Changchun, China

**Keywords:** DDX3X, interactome, RK-33, rotavirus, VP4

## Abstract

Rotavirus (RV) is a significant zoonotic pathogen primarily causing severe diarrheal disease in humans and animals, posing substantial risks to global public health and livestock industries. VP4 is one of the outer capsid proteins of RV and plays a crucial role in RV attachment and internalization. Additionally, it is also involved in replication and immune responses during RV infection; however, related studies are still limited. Here, a comprehensive analysis of the RV VP4 interactome was conducted, and DDX3X, one of six DExD/H helicase family members identified as interacting with VP4, potentially plays a crucial role in RV infection. Silencing DDX3X inhibits RV infection, whereas its overexpression facilitates RV infection. Further research demonstrated that VP4 interacts with DDX3X and the enzymatic activity of DDX3X was found to contribute to promote RV replication. Additionally, a drug screening study based on the VP4 interactome identified RK-33, a potent inhibitor of DDX3X, as the most effective candidate compound for inhibiting RV. In conclusion, VP4 interacts with DDX3X and the enzymatic activity of DDX3X is crucial for RV replication. The DDX3X inhibitor RK-33 exhibits significant inhibitory effects on RV infection. This study highlights the important roles of DDX3X in RV infection, offering potential candidate drugs for RV and expanding our understanding of its mechanisms.

## 1. Introduction

Rotavirus (RV) disease primarily manifests as RV gastroenteritis, an acute and highly contagious zoonotic pathogen [1]. The primary symptoms include severe diarrhea, vomiting, and death in infants and young animals. Additionally, RV can also cause extraintestinal symptoms such as biliary atresia, central nervous system damage, respiratory system damage, heart damage, et cetera [1–5]. Annually, it imposes a serious medical burden on humans and significant economic losses on the livestock industry. RV are characterized by their wide transmission range, strong mutational capacity, and high difficulty in prevention and control, making them a significant subject of public health and basic research [6, 7].

RV belongs to the family Reoviridae, a nonenveloped and double-stranded RNA virus, and consists of 11 genome segments. It has three layers of capsid proteins: the core of RV is composed of VP1, VP2, and VP3; the middle layer is the VP6; the outer layer comprises VP4 and VP7. VP4 is the outermost protein of RV and plays an important role in mediating viral entry, replication, and immunity [8–11]. While the role of VP4 in viral entry has been extensively studied, few investigations have focused on its functions beyond this step.

A proximity labeling method utilizing a promiscuous biotin ligase gene, known as TurboID, enables covalent labeling of neighboring proteins with biotin within 10 min. This method has been successfully applied to various protein–protein interaction (PPI) studies [12–17]. MYAS33 RV strain is a Type A RV isolated from bats [18], and we have demonstrated that it can infect various animal cell lines and conducted VP4 interaction identification using TurboID [19]. Through analysis of our VP4 interactome data, it suggests that VP4 interacting proteins have functions enriched not only in viral entry but also in replication and immunity. Therefore, we aim to uncover important host proteins from the VP4 interactome beyond mediating viral entry.

According to our VP4 interactome dataset, 174 host proteins were identified, including six members of the DExD/H (aspartic–glutamate-x-aspartic/histidine) helicase family, which are closely related to innate immunity and viral replication. Among them, DDX3X was found to have the most biotin labeled sites, prompting a series of investigations to elucidate its role in RV infection and deciphering the underlying mechanisms. Additionally, based on the VP4 interactome, various candidate anti-RV drugs were screened and identified. RK-33, an inhibitor of DDX3X, exhibited the strongest anti-RV effect and was further explored both *in vitro* and *in vivo*. These findings have expanded our understanding of RV–host interactions and provided novel theoretical insights for the development of antiviral drugs.

## 2. Results

### 2.1. RV VP4 Interacts With DDX3X

**Figure 1:**
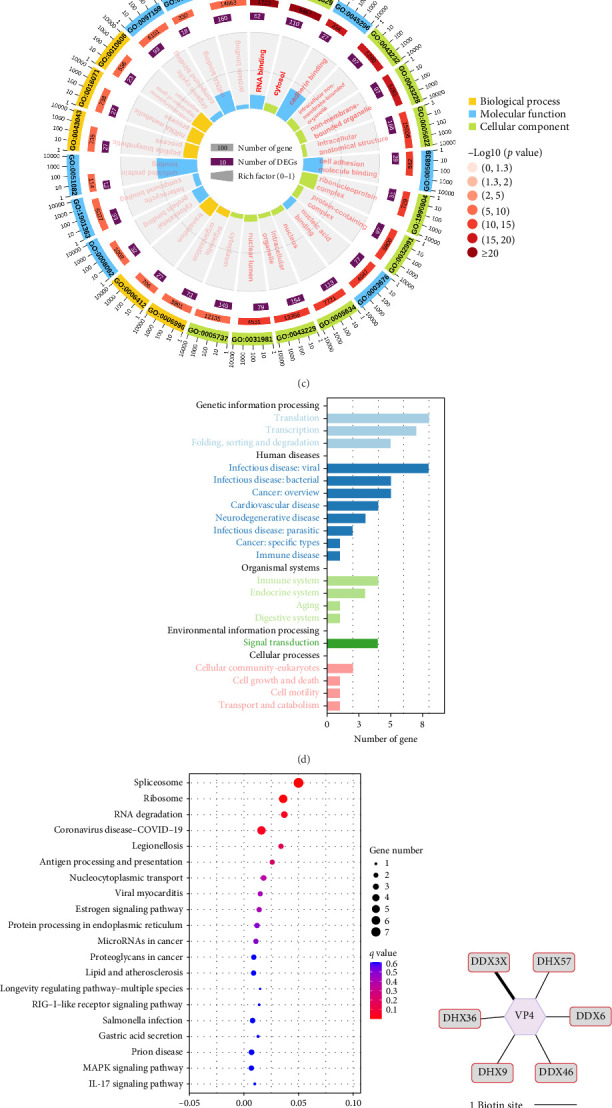
RV VP4 interacts with DDX3X. (a) MYAS33 RV strain viral protein expression plasmids and controls were transfected, respectively, then, stimulated by poly (I:C), and detected the relative expression levels of IFNB1 and IFNL1. (b) VP4 interacting protein classified by families and display families with four or more members. (c) Comprehensive GO enrichment of biological process, molecular function, and cellular component for VP4 interacting proteins. (d, e) Perform pathway and functional enrichment for RNA binding related genes. (f) Number of biotin sites labeled in the DExD/H-box helicase family. (g) Transfected with Myc-DDX3X, HA-VP4, or empty vectors, then applied Myc conjugated magnetic beads for immunoprecipitation and detected by western blot. (h) Transfected with Myc-DDX3X and HA-VP4, fixed and blocked, incubated with Myc and HA antibodies, then, incubated with FITC and CY3 conjugated secondary antibodies, respectively, and applied DAPI to stain DNA, followed by confocal detection.

VP4 is the outermost protein of RV, primarily mediating viral entry, while research on its other functions remains limited. Several studies have shown that VP4 is also involved in viral replication and immunity. MYAS33 RV strain is a Group A RV, and we have demonstrated that it can infect various animal cell lines [18, 19]. In addition, we evaluated the inhibitory effects of each MYAS33 protein on interferon in Caco-2 and found that several viral proteins, including VP3, VP4, nsp1, nsp3, and nsp4, significantly suppressed interferon beta 1 (IFNB1) or IFN lambda 1 (IFNL1) expression especially nsp1, VP3, and VP4 (Figure 1a). Among these, nsp1 and VP3 have been extensively studied for their roles in antagonizing interferon responses. Therefore, we became interested in the functions of VP4 beyond mediating viral entry.

To investigate potential key host proteins, VP4 interacting proteins identified by proximity labeling were classified into families and there are four families each containing four or more members (Figure 1b). These families include the DExD/H-box helicase family, the ribosomal protein family, the heat shock protein family, and the eukaryotic translation initiation factor family. Notably, the DExD/H-box helicase family exhibited the most interacting members, which is particularly noteworthy given its crucial role in innate immunity and viral replication.

Additionally, enrichment analysis of VP4 interactions revealed “RNA binding” as the most significant functional category. Furthermore, pathway enrichment analysis of RNA binding proteins highlighted their potential roles in antiviral defense and viral replication (Figure 1c–e), further pointing to the involvement of the DExD/H-box helicase family.

The six members of the DExD/H-box helicase family identified as VP4 interacting proteins included DDX3X, DDX46, DDX6, DHX9, DHX57, and DHX36. Among these, DDX6 has been reported to promote RV infection [20]. Further analysis indicated that DDX3X was uniquely marked at three biotin sites within the VP4 proximity labeling dataset, whereas the other five helicase family members were each labeled with a single site (Figure 1f). Subsequently, the interaction between VP4 and DDX3X was examined by co-immunoprecipitation (Co-IP) assay. The results showed that VP4 could interact with DDX3X (Figure 1g). Additionally, a confocal assay determined that VP4 and DDX3X colocalized (Figure 1h).

### 2.2. DDX3X Promotes RV Infection

**Figure 2:**
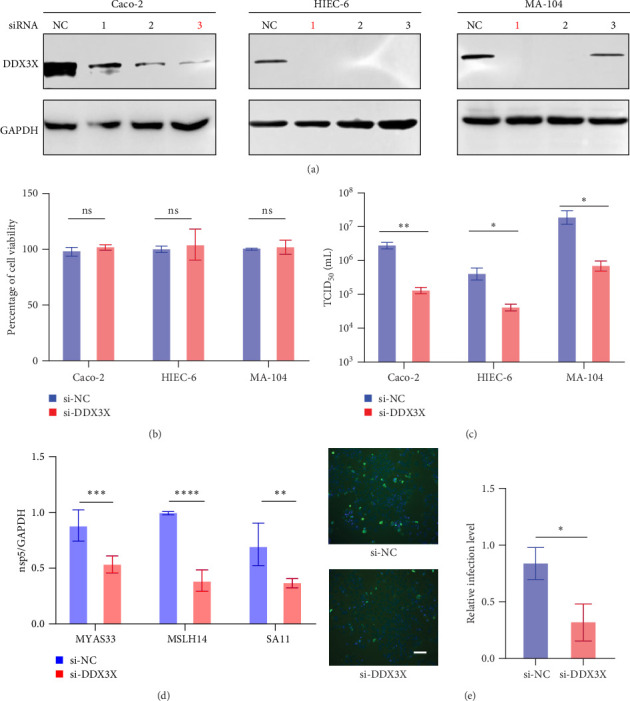
Knockdown of DDX3X restricts RV infection. (a) HIEC-6, Caco-2, and MA-104 were transfected with DDX3X siRNAs and negative control siRNA, respectively, then verify the knockdown efficiency of each siRNA by western blot. (b) HIEC-6, Caco-2, and MA-104 were transfected with DDX3X siRNA and negative control siRNA separately, then, evaluate cell viability using the CCK-8 method. (c) HIEC-6, Caco-2, and MA-104 were transfected with DDX3X siRNA and negative control siRNA, respectively, then infected with MYAS33 RV strain, and 24 h later, examined viral titer using the TCID_50_ method. (d) After knocking down DDX3X, infected the cells with MYAS33, MSLH14, and SA11 RV strains, respectively; 24 h later, collected the cells and detected the relative expression levels of RV nsp5 by RT-qPCR. (e) DDX3X siRNA and negative control siRNA were transfected, respectively, followed infected with RV. Cells were fixed, permeabilized, incubated with VP6 antibody as the primary antibody, and FITC-conjugated secondary antibody was used for detection. Fluorescence microscopy was used to observe the results, the scale bar represents 100 μm. Statistical significance is determined by Student's test (*⁣*^*∗*^*p* < 0.05; *⁣*^*∗∗*^*p* < 0.01; *⁣*^*∗∗∗*^*p* < 0.001; *⁣*^*∗∗∗∗*^*p* < 0.0001; ns, not significant).

To investigate the role of DDX3X in RV infection, knockdown of DDX3X was performed, and the most effective siRNA was selected in Caco-2, HIEC-6, and MA-104 cells (Figure 2a). A cell counting kit-8 (CCK-8) assay showed no significant changes in cell viability after siRNA transfection (Figure 2b). Subsequently, the cells were infected with the MYAS33 RV strain, and virus titers were measured at 24 h postinfection (hpi). As shown in Figure 2c, compared with siNC (negative control), knockdown of DDX3X significantly reduced virus titer in Caco-2, HIEC-6, and MA-104 cells, indicating that DDX3X could facilitate RV infection.

In addition to MYAS33, we also investigated the effect of knocking down DDX3X on MSLH14 and SA11 RV strains and examined the relative expression levels of the nsp5 gene at 24 hpi using real time quantitative polymerase chain reaction (RT-qPCR). The results showed that knockdown of DDX3X significantly restricted infection by all these RV strains (Figure 2d). And the immunofluorescence assay also demonstrated that knocking down DDX3X restricted RV infection (Figure 2e).

**Figure 3:**
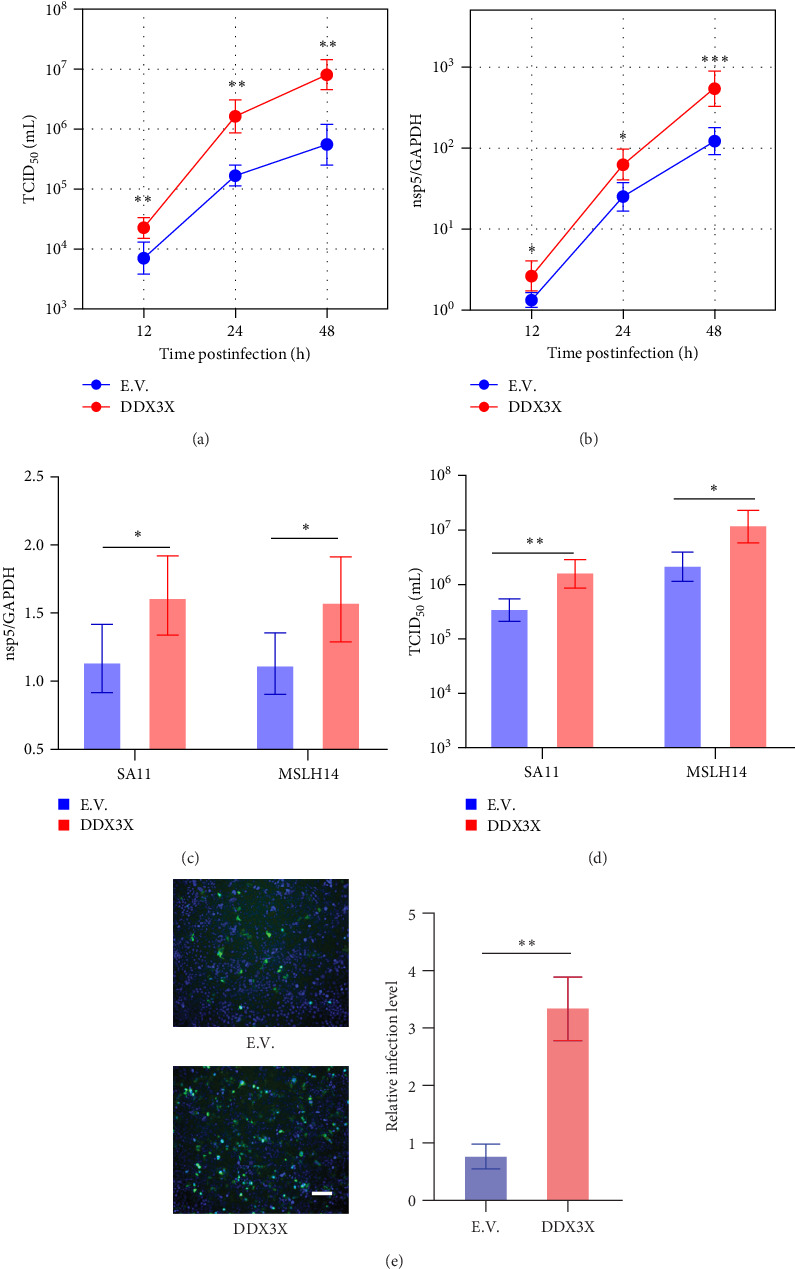
Overexpression of DDX3X promotes RV infection. (a, b) DDX3X or empty vector (E.V.) were transfected, respectively, then infected with MYAS33 RV strain, and performed relative quantification and TCID_50_ detection at 12, 24, and 48 h. (c, d) After overexpressing DDX3X, infected the cells with SA11 and MSLH14 RV strains, respectively, detected RV nsp5 expression by RT-qPCR and examined viral titer. (e) DDX3X or E.V. were transfected, followed infected with RV. Cells were fixed, permeabilized, incubated with VP6 antibody as the primary antibody, and FITC-conjugated secondary antibody was used for detection. Fluorescence microscopy was used to observe the results, the scale bar represents 100 μm. Statistical significance is determined by Student's test (*⁣*^*∗*^*p* < 0.05; *⁣*^*∗∗*^*p* < 0.01; *⁣*^*∗∗∗*^*p* < 0.001; *⁣*^*∗∗∗∗*^*p* < 0.0001; ns, not significant).

To determine whether overexpressing DDX3X could enhance RV infection, Caco-2 cells were transfected with either DDX3X or an empty vector and infected with MYAS33. Virus titers and nsp5 gene expression levels of RV were examined at 12, 24, and 48 hpi. The results demonstrated that overexpression of DDX3X significantly promoted RV infection at all tested time points for both virus titer (Figure 3a) and nsp5 levels (Figure 3b). Additionally, overexpressing DDX3X also significantly increased SA11 or MSLH14 RV strain infections at 24 hpi in terms of both gene levels (Figure 3c) and virus titers (Figure 3d). Additionally, the immunofluorescence assay also suggested overexpression DDX3X promoted RV infection (Figure 3e). All these findings collectively demonstrate that DDX3X promotes RV infection.

### 2.3. VP4 Inhibits DDX3X Induced Interferon Production in Caco-2 Cells

**Figure 4:**
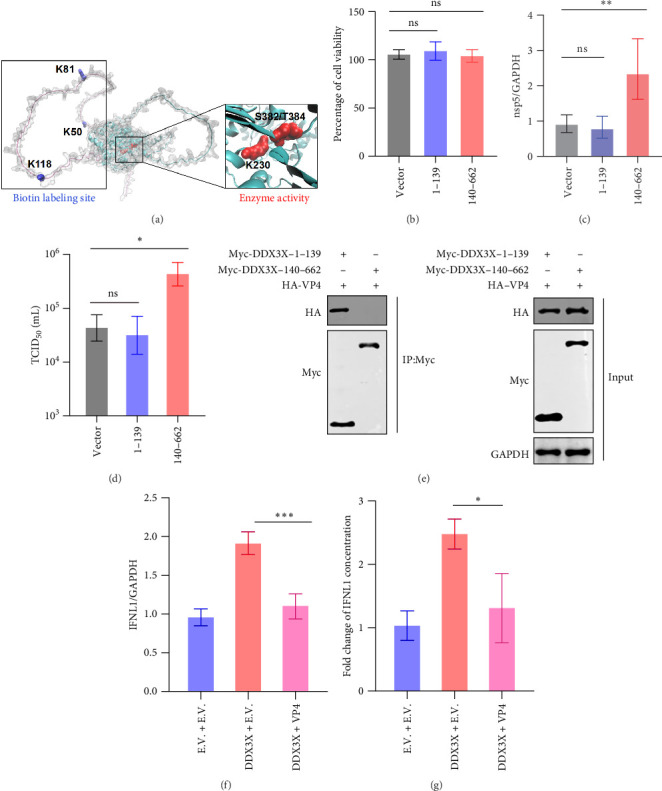
VP4 inhibits DDX3X induced interferon expression. (a) Structure of DDX3X. (b) The effect of transfection on cell viability was determined by CCK-8 method. (c, d) DDX3X 1–139 aa, 140–662 aa, or empty vector were transfected, followed by infection with MYAS33 RV strain. The viral detection was conducted 24 hpi by RT-qPCR and TCID_50_. (e) Identification of the interaction regions between VP4 and DDX3X: DDX3X 1–139 aa, 140–662 aa, and VP4 were transfected, respectively, then use Myc conjugated magnetic beads for immunoprecipitation and detected by western blot. (f, g) VP4 inhibits DDX3X-induced interferon expression in Caco-2. Statistical significance is determined by Student's test (*⁣*^*∗*^*p* < 0.05; *⁣*^*∗∗*^*p* < 0.01; *⁣*^*∗∗∗*^*p* < 0.001; *⁣*^*∗∗∗∗*^*p* < 0.0001; ns, not significant).

According to the VP4 proximity labeling dataset, three amino acids (aas) were labeled with biotin, and all were located within the N-terminal region (1–139 aa), which is known as the interferon induced domain, while the 140–662 aa region contains the enzymatic activity domain (Figure 4a). We aimed to investigate which region plays a crucial role in DDX3X promoting RV infection.

The two regions were overexpressed in Caco-2 cells separately and showed no significant changes in cell viability after transfection (Figure 4b). RV was infected post transfection, and subsequent examination of nsp5 relative expression and virus titer. The results revealed that overexpression of the DDX3X (1–139 aa) had no significant effect on RV infection, whereas overexpression of the DDX3X (140–662 aa) promoted RV infection (Figure 4c,d).

The 1–139 aa region of DDX3X is an interferon induced domain that might have a function to inhibit RV infection but showed no effect on RV infection in this study. Notably, all three biotin labeled sites in DDX3X were located within the 1–139 aa region, suggesting that VP4 might interact with the 1–139 aa of DDX3X to exert its inhibitory effect. To confirm which region of DDX3X interacts with VP4, Co-IP assays were performed. The results demonstrated that VP4 interacted with the 1–139 aa region of DDX3X, but not with the 140–662 aa region (Figure 4e). Furthermore, additional experiments in Caco-2 cells showed that co-transfection of DDX3X and VP4 plasmids resulted in significantly reduced IFNL1 expression compared to cells transfected with DDX3X alone, indicating that VP4 interferes with DDX3X induced interferon expression (Figure 4f,g).

### 2.4. The Enzymatic Activity of DDX3X Plays a Crucial Role in RV Replication

**Figure 5:**
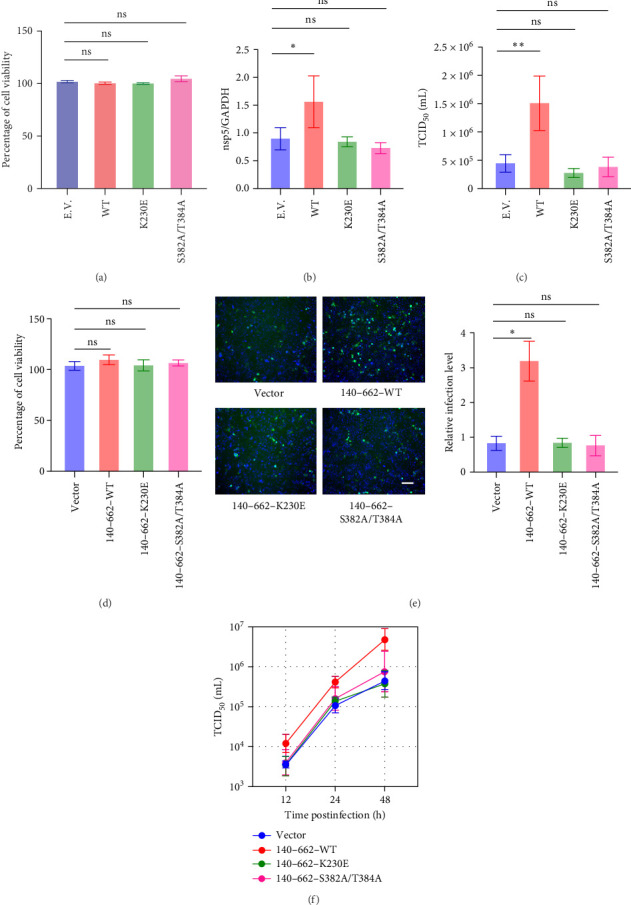
Enzymatic activity of DDX3X promotes RV replication. (a) DDX3X (140–662 aa) or its K230E and S382A/T384A mutants were transfected and the cell viability was evaluated by CCK-8 method. (b, c) Wild type DDX3X, K230E mutant, or S382A/T384A mutant and empty vector were transfected, respectively, followed infected with RV, the viral detection was conducted 24 hpi by RT-qPCR and TCID_50_. Cell viability was measured by CCK-8 (d). (e) Cells were infected with RV, and 24 hpi cells were fixed, permeabilized, incubated with VP6 antibody as the primary antibody, and FITC-conjugated secondary antibody was used for detection. Fluorescence microscopy was used to observe the results, the scale bar represents 100 μm. (f) DDX3X 140–662 aa or K230E and S382A/T384A mutants were transfected, respectively, followed infected with RV. The viral detection was conducted 24 hpi by TCID_50_. Statistical significance is determined by Student's test (*⁣*^*∗*^*p* < 0.05;*⁣*^*∗∗*^*p* < 0.01; *⁣*^*∗∗∗*^*p* < 0.001; *⁣*^*∗∗∗∗*^*p* < 0.0001; ns, not significant).

As the region of DDX3X that promotes RV infection is located within the 140–662 aa region, which contains the enzymatic activity domain, this study further investigated whether the enzymatic activity of DDX3X plays a role in RV infection. The K230 residue, as well as the S382/T384 residues, are critical for the enzymatic activity of DDX3X [21]. These sites were individually mutated, and the CCK-8 assay showed no significant changes in cell viability after transfection (Figure 5a). Infection experiments showed that overexpression of wild type DDX3X significantly promoted RV infection, while the K230E mutation and the S382A/T384A mutations exhibited no significant proviral effect on RV infection at both transcript levels (Figure 5b) and virus titer (Figure 5c), indicating that the enzymatic activity domain of DDX3X plays a crucial role in effective viral replication.

To further confirm this finding, the K230 residue, as well as the S382/T384 residues, were mutated within the 140–662 aa region of DDX3X, thereby eliminating the influence of the N-terminal. A CCK-8 assay showed no significant changes in cell viability after transfection (Figure 5d). Immunofluorescence results showed that wild-type 140–662 aa promoted RV infection, whereas the mutants did not (Figure 5e). Viral titer assays further demonstrated that the wild type 140–662 aa promoted RV infection at 12, 24, and 48 hpi, while the mutants showed no such effect (Figure 5f). These results conclusively demonstrate that the enzymatic activity domain of DDX3X plays a critical role in RV replication.

### 2.5. Drug Screening Based on Interactome Demonstrated That the DDX3X Inhibitor RK-33 Exhibited the Best Inhibitory Effect on RV Infection

Systematic studies of viral protein interactions not only contribute to investigating crucial interacting proteins but also play a significant role in the discovery of potential antiviral drugs based on the interactome. Consequently, this study investigated potential anti-RV drugs based on VP4 interacting proteins.

**Figure 6:**
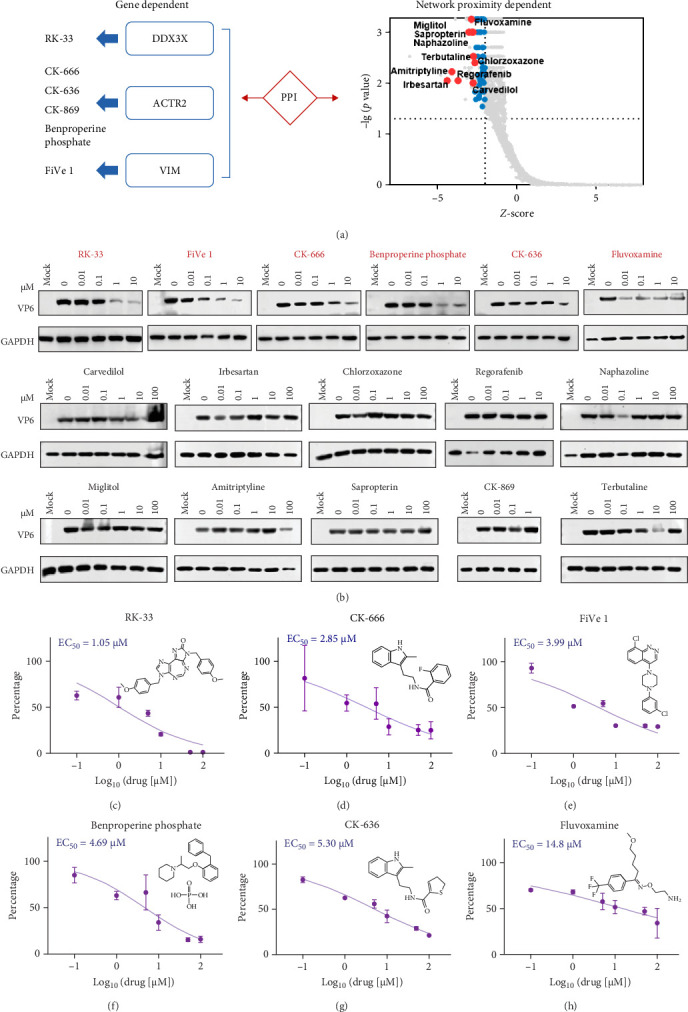
Drug screening based on interactome shows that DDX3X inhibitor RK-33 with best effect on inhibition of RV infection. (a) Drugs were selected by gene dependent and network dependent strategy based on VP4 interacting proteins. (b) Cells were treated with a range of drug concentrations, and then, infected with MYAS33 RV strain. Subsequently, the cells were collected and subjected to western blot analysis to detect viral proteins. (c–h) EC_50_ was determined for the screened drugs that showed anti-RV activity.

The candidate drug selection was approached from two distinct strategy (Figure 6a). First, we utilized a gene dependent drug selection strategy, targeting key interacting proteins known to play critical roles in RV infection. The second approach employed a network based drug selection method, which comprehensively considers the interactome of proteins and has been previously validated and reported [22].

For the gene dependent drug selection, aside from RK-33, an inhibitor specifically targeting DDX3X, drugs associated with VIM and ACTR2, which we have previously demonstrated play pivotal roles during RV infection, was also selected [19]. In the network-based approach, thousands of FDA approved drugs were evaluated and scored. From this evaluation, the top 10 small molecule candidates were selected for further investigation.

Subsequently, these candidate drugs underwent cell viability inhibition test (Figure S1) and then was screened the antiviral efficacy (Figure 6b). The results revealed that six of these compounds, RK-33, CK-666, FiVe 1, benproperine phosphate, CK-636, and fluvoxamine exhibited inhibitory effects against RV infection. Furthermore, EC_50_ determinations were conducted, and RK-33 displayed the lowest EC_50_ of 1.05 μM, while the EC_50_ of CK-666, FiVe 1, benproperine phosphate, CK-636, and fluvoxamine was 2.85, 3.99, 4.69, 5.30, and 14.8 μM, respectively, indicating its potent antiviral activity compared to the other candidate drugs (Figure 6c).

### 2.6. The Inhibition of RV Infection by RK-33

RK-33 is a specific inhibitor of DDX3X and demonstrated the strongest antiviral effect among the candidate compounds. The inhibitory effects of RK-33 against RV were further investigated both in vitro and in vivo.

**Figure 7:**
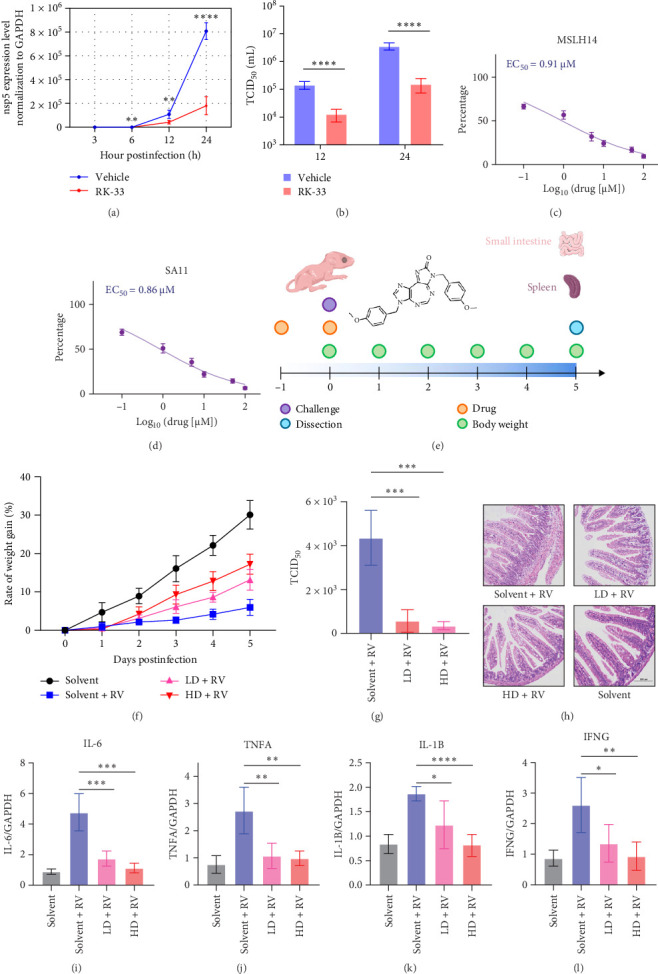
RK-33 inhibits RV infection. (a, b) Cells were incubated with 10 μM RK-33 and infected with MYAS33 RV strain. The viral detection was conducted at 3, 6, 12, and 24 hpi by RT-qPCR detection, and at 12 and 24 hpi by TCID_50_. (c, d) EC_50_ of RK-33 against MSLH14 and SA11 strains was measured. (e) Flowchart of in vivo drug experiment. (f) Weight gain rate of mice. (g) Intestinal virus titer detection in mice. (h) H&E staining of intestine. (i–l) Cytokine detection of spleen. Statistical significance is determined by Student's test (*⁣*^*∗*^*p* < 0.05; *⁣*^*∗∗*^*p* < 0.01; *⁣*^*∗∗∗*^*p* < 0.001; *⁣*^*∗∗∗∗*^*p* < 0.0001). HD, high dose; LD, low dose; ns, not significant.

To assess its antiviral activity in vitro, cells were treated with RK-33 and infected with the MYAS33 RV strain. Samples were collected at 3, 6, 12, and 24 hpi for RT-qPCR analysis. The results showed that RK-33 had no significant inhibitory effect on RV at 3 hpi, but exhibited pronounced antiviral activity at 6, 12, and 24 hpi (Figure 7a). Additionally, viral titer examinations of the 12 and 24 h samples consistently demonstrated that RK-33 inhibited MYAS33 strain infection (Figure 7b). Subsequent experiments were conducted to evaluate the ability of RK-33 to inhibit two additional RV strains, SA11 and MSLH14, and the results suggested that RK-33 also inhibits SA11 and MSLH14 infections (Figure 7c,d). These findings further confirmed that RK-33 exhibits significant anti-RV activity in vitro.

For *in vivo* experiment, mice were treated with RK-33, with a solvent applied as a control and challenged with RV (Figure 7e). RK-33 treated animals exhibited faster recovery of body weight compared to untreated controls, and viral titers were significantly reduced in the RK-33-treated groups relative to untreated controls (Figure 7f,g). Histopathological analysis of intestinal tissues at 5 days postinfection revealed that RK-33 treatment alleviated intestinal lesions (Figure 7h). To assess inflammatory responses, spleen tissues were analyzed for cytokine levels. Compared to untreated controls, RK-33 treated animals showed significantly reduced levels of interleukin 6 (IL-6), tumor necrotic factor alpha (TNFA), IL 1 beta (IL-1B), and IFN gamma (IFNG), suggesting that RK-33 treatment substantially lowered inflammatory cytokine expression (Figure 7i–l). This study demonstrates that RK-33 reduces intestinal viral loads and inflammation levels. Collectively, these experiments provide evidence that RK-33 inhibits RV infection both *in vitro* and *in vivo*.

## 3. Discussion

RV is an important zoonotic virus that primarily causes severe gastroenteritis, posing a significant threat to human health and animal husbandry. RV is a segmented RNA virus with a three layered capsid, and the outer layer consists of VP4 and VP7 proteins. The primary function of the VP4 protein is to mediate the viral entry process. Additionally, it also participates in viral replication and immunity; however, research in this area remains limited.

Analysis of our VP4 interactome dataset demonstrates that proteins interacting with VP4 are functionally enriched in processes such as viral entry, replication, and immunity. Furthermore, the effect of each MYAS33 protein on interferon expression was investigated, and it was found that VP3, VP4, nsp1, nsp3, and nsp4 exhibited inhibitory effects on IFNB1 or IFNL1 expression, particularly nsp1, nsp3, and VP4. Both the VP4 interactome analysis and experimental results suggest functions beyond viral entry.

Family analysis of VP4 interacting proteins revealed that the family with the most members is the DExD/H-box family, which is closely related to antiviral innate immunity and viral replication. The identified DExD/H-box family members include DDX3X, DDX46, DDX6, DHX9, DHX57, and DHX36. Among these, DDX3X was labeled with the most biotin sites, indicating potential stronger binding.

The role of DDX3X in viral infection is complex and multifaceted. Initially identified for its ability to stimulate interferon production and suppress viral infections, subsequent studies have revealed that DDX3X can also promote the replication of certain viruses, including West Nile virus, hepatitis C virus, SARS-CoV-2, herpes simplex virus Type 1, HIV, and Japanese encephalitis virus [21, 23–29].

Our research demonstrates that DDX3X interacts with VP4 and facilitates RV infection. Furthermore, VP4 inhibits the interferon inducing activity of DDX3X, and the enzymatic region of DDX3X plays a crucial role in this process. On the other hand, based on the VP4 interacting network, we identified candidate drugs using gene dependent and network-dependent approaches. After screening, we found that RK-33, CK-636, CK-666, benproperine phosphate, FiVe 1, and fluvoxamine exhibit inhibitory effects, with RK-33 being the most effective. RK-33 is a K230 site inhibitor of DDX3X, a residue proven in this study to be critical for RV infection. Further research confirms that RK-33 can effectively suppress RV infection both in vitro and in vivo.

In summary, this study, based on VP4 interactome research, found that DDX3X interacts with VP4, suppresses its interferon inducing effect in Caco-2 cells, while its enzymatic activity region promotes RV infection. The DDX3X enzymatic activity inhibitor RK-33 exhibits inhibitory effects against RV both in vitro and in vivo, providing a theoretical foundation for further drug development.

## 4. Materials and Methods

### 4.1. Cells and Viruses

HIEC-6, MA-104, Caco-2, and 293T cells were maintained in our laboratory. All cells were cultured in Dulbecco's modified eagle's medium (Gibco) containing 10% fetal bovine serum (Gibco) and 1% penicillin-streptomycin solution (Hyclone).

RV strains MYAS33 and MSLH14 were kindly provided by Prof. Biao He, and SA11 was kindly provided by Yuzhang Wu. The MYAS33 and MSLH14 were propagation in Marc-145 cells and the SA11 were propagation in MA-104 cells as previously described [18, 30, 31].

### 4.2. Antibodies and Reagents

DDX3X antibody were purchased from Proteintech. RV VP6 antibody was purchased from Santa Cruz Biotechnology. Antibodies against GAPDH and Myc were purchased from Cell signaling Technology. HA Tag rabbit polyclonal antibody, horseradish peroxidase (HRP) labeled goat anti-rabbit IgG (H + L), HRP-labeled goat anti-mouse IgG (H + L), Cy3 labeled goat anti-rabbit IgG (H + L), and FITC labeled goat anti-mouse IgG (H + L) antibodies were purchased from Beyotime Institute of Biotechnology. Carvedilol, irbesartan, chlorzoxazone, naphazoline, regorafenib, miglitol, amitriptyline, sapropterin, terbutaline, and fluvoxamine were purchased from Topscience Co. Ltd. FiVe 1, CK-666, benproperine phosphate, CK-636, and CK-869 were purchased from MedChemExpress, and RK-33 purchased from ApexBio Technology. The human lambda 1 ELISA Kit was purchased from Sinobiological.

Lipofectamine RNAiMAX and Lipofectamine 3000 were purchased from Invitrogen.

### 4.3. Plasmids, siRNA, and Transfection

Human DDX3X coding sequences were amplified and then cloned to pcDNA3.1-3Myc vector, the mutants were constructed by Sangon Biotech. Plasmid transfection was performed with the procedure carried out according to the manufacturer's instructions. The siRNAs used in this study were purchased from Ribobio Biotechnology, and all transfections were conducted following the manufacturer's protocol.

### 4.4. Western Blot

Cells were collected, washed with PBS, and digested with trypsin. The cells were then collected in PBS into centrifuge tubes. After centrifugation, the supernatant was discarded. The cells was lysed, and the resulting lysate was transferred to a new centrifuge tube. 5 × SDS-PAGE sample buffer was added, and the samples were boiled in a water bath. Subsequently, SDS-PAGE was performed, and the proteins were transferred onto nitrocellulose membranes. The membranes were blocked with 5% skim milk at room temperature, washed three times with TBST, and incubated overnight at 4°C with primary antibodies. After washing three times with TBST, corresponding secondary antibodies were added and incubated for 1 h at room temperature. Following three additional washes with TBST, ECL reagent was prepared and applied to the membrane. Finally, the membrane was exposed in a chemiluminescence imaging system.

### 4.5. IP

293T cells were seeded into a six-well plate at a density of 1 × 10^6^ cells per well. Once adherent, the cells were transfected with their respective plasmids and incubated for 48 h before harvesting. The cells were lysed on ice using 500 μL of IP lysis buffer. After centrifugation, the supernatant was collected. A 50 μL sample was combined with 5 × SDS sample buffer and boiled at 95°C for 10 min to prepare the input samples. The remaining lysate was used for IP. Myc antibody-conjugated magnetic beads were added, and the mixture was incubated overnight at 4°C. After washing the magnetic beads five times with IP lysis buffer, they were resuspended in 5 × SDS sample buffer and boiled at 95°C for 10 min. Subsequent western blot analysis was performed using both the input and IP samples.

### 4.6. Immunofluorescence and Confocal Assay

The cells were washed with PBS, 4% paraformaldehyde was then added for fixation at room temperature for 15–20 min. Following three washes with PBS, 0.1% Triton X-100 was used for permeabilization, followed by an additional three washes with PBS. Subsequently, a 3% BSA solution was applied for blocking, and the cells were washed again with PBS. Primary antibody was then added, and the samples were incubated at 4°C overnight. After washing three times with PBS, corresponding secondary antibody was applied, followed by a 1 h incubation. Finally, the cells were washed three times with PBS before being imaged on a fluorescence microscope.

### 4.7. RNA Isolation and RT-qPCR Analysis

Total RNA was extracted from collected samples using TRIzol reagent (Sangon Biotech) and subsequently reverse transcribed into cDNA using a premix reverse transcriptase (Vazyme). For qPCR analysis, a SYBR Green qPCR Mix kit (Promega) was utilized in combination with a CFX96 system. All procedures were carried out following the manufacturers' guidelines as described previously [19]. The specific primers used to RT-qPCR were listed in Table 1.

### 4.8. Antiviral Study In Vitro and Vivo

Cells were seeded into 96-well plates, allowed to adhere, followed by drug inoculation in a concentration gradient along with RV infection at 0.1 MOI. After 24 h, cells were collected, total RNA was extracted, reverse transcription was performed, and RV was detected by RT-qPCR. The EC_50_ of the drug was calculated.

Pregnant Kunming mice were individually housed and bred until delivery. Neonatal mice were raised for 1 week. Mice were inoculated with 10^4^ TCID_50_ units of the MSLH14 RV strain via gavage. Drug administration was performed with 0.005 mg/g (low dose group) or 0.01 mg/g (high dose group). The drugs were dissolved in a solvent formulated as follows: 10% DMSO, 40% PEG300, 5% Tween-80, and 45% normal saline. Viral titers in the small intestine were determined using the TCID_50_ method. Spleens were collected for total RNA extraction, followed by reverse transcription and cytokine detection. For histopathological examination, tissues were fixed with 4% paraformaldehyde, paraffin embedded, sectioned into slices, and stained with hematoxylin and eosin following standard protocols as previously described [2, 32].

All animal experiments were approved by the ethics committee of Research Unit of Key Technologies for Prevention and Control of Virus Zoonoses, Chinese Academy of Medical Sciences, Changchun Veterinary Research Institute, and Chineses Academy of Agricultural Sciences (IACUC of AMMS-11-2023-018).

### 4.9. Statistical Analysis

The statistical significance between groups was assessed using GraphPad Prism software. All data are presented as mean ± standard deviation (SD). Statistical significance was determined by two-tailed Student's *t*-test (for comparisons between two groups). *p* values less than 0.05 were considered significant, and significant differences are indicated in the figures as follows: *⁣*^*∗*^*p* < 0.05; *⁣*^*∗∗*^*p* < 0.01; *⁣*^*∗∗∗*^*p* < 0.001; *⁣*^*∗∗∗∗*^*p* < 0.0001; ns, not significant.

## Figures and Tables

**Table 1:** Primers used in this study.

Primer	Sequence (5′–3′)
GAPDH-F	CTACATGGTTTACATGTTCC
GAPDH-R	GGATCTCGCTCCTGGAAGAT
nsp5-F	CGTCAACTCTTTCTGGAAAATC
nsp5-R	GCATTTGTCTTAACTGCATTCG
IFNL1-F	TTCCAAGCCCACCACAACTG
IFNL1-R	GAGTGACTCTTCCAAGGCGT
IFNB1-F	CAGTGTCAGAAGCTCCTGTGGC
IFNB1-R	CATAGATGGTCAATGCGGCGTC
IFNG-F	AGTCTCTTCTTGGATATCTGG
IFNG-R	ATGACGCTTATGTTGTTGCTG
TNFA-F	CTCAAAGACAACCAACTAGTG
TNFA-R	TGGTATGAGATAGCAAATCGG
IL-6-F	CACTTCACAAGTCGGAGGCT
IL-6-R	CTGCAAGTGCATCATCGTTGT
IL-1B-F	TGTGGAGAAGCTGTGGCAG
IL-1B-R	CAGCAGGTTATCATCATCATC

## Data Availability

The data of VP4 interactome analyzed in this study were deposited in iProX with accession number IPX0007242000, and the other data are available in the manuscript and supporting information.

## Supporting Information

Supporting Information Figure S1: Identification of drug inhibition on cell viability. (A–P) Cell viability was measured using the CCK-8 assay after drug treatment.
